# Navigating the Intersection of Heart Failure and Palliative Care: A Holistic Approach to Improving Quality of Life

**DOI:** 10.7759/cureus.81466

**Published:** 2025-03-30

**Authors:** Awanwosa V Agho, Fatimot Disu, Efeturi M Okorigba, Okelue E Okobi, Safiyya Muhammad, Toheeb Bakare, Chioma Ezuma-Ebong, Nneka Muoghalu

**Affiliations:** 1 Internal Medicine, Mercy Catholic Medical Center, Darby, USA; 2 General Internal Medicine, Salisbury National Health Service (NHS) Foundation Trust, Salisbury, GBR; 3 Internal Medicine, West Virginia University, Morgantown, USA; 4 Family Medicine, IMG Research Academy and Consulting, Homestead, USA; 5 Family Medicine, Larkin Community Hospital Palm Springs Campus, Hialeah, USA; 6 Family Medicine, Lakeside Medical Center, Belle Glade, USA; 7 General Practice, Mersey and West Lancashire Teaching Hospitals National Health Service (NHS) Trust, Rainhill, GBR; 8 Internal Medicine/Neurology/Cardiology, Southmead Hospital, Bristol, GBR; 9 Internal Medicine, Interfaith Medical Center, New York City, USA; 10 Public Health, University of Liverpool, School of Tropical Medicine, Liverpool, GBR; 11 Internal Medicine, University College Hospital, Ibadan, NGA

**Keywords:** advance care planning, heart failure, palliative care, quality of life, symptoms burden

## Abstract

Heart failure (HF) is a multifaceted, severe condition linked to significant emotional, physical, and social challenges that have an immense impact on the patient’s quality of life. Notwithstanding the advancements in medical treatment, many HF patients experience recurrent hospitalizations, gradual functional decline, and various incapacitating symptoms. The integration of palliative care into the management of HF offers a holistic approach that addresses not only the physical symptoms but also the emotional, psychological, and spiritual needs of patients, their families, and caregivers. The integration of palliative care in HF management addresses the patients’ holistic requirements, which enhances the quality-of-life outcomes for the patients through the provision of emotional and psychological support and aiding caregivers in managing the challenges associated with HF. Regardless of the acknowledged advantages of integrating PC into heart failure management, execution has largely remained poor in most healthcare services globally. The objective of this systematic review is to identify how the integration of palliative care into heart failure treatment interventions improves the quality of life for heart failure patients. To attain the set objective, we conducted an extensive search on references drawn from diverse online databases, such as Embase, PubMed, SCOPUS, Web of Science, and Google Scholar. Cross-over design studies randomized controlled trials (RCTs), systematic reviews and meta-analyses, and prospective cohort studies that focused on palliative care in heart failure patients were selected and subsequently included. From the search, 18 studies satisfied the inclusion criteria and were consequently included following evaluations using the preferred reporting items for systematic reviews and meta-analyses (PRISMA) guidelines. The review disclosed that PC is effective in improving the quality of life (QoL) of heart failure patients.

## Introduction and background

According to the American Heart Association (AHA) guidelines, heart failure (HF) is defined as a complex clinical syndrome resulting from any structural or functional impairment of ventricular filling or ejection of blood [1,2]. This means the heart cannot meet the body's metabolic demands due to systolic or diastolic dysfunction. HF is categorized based on ejection fraction (EF) into heart failure with reduced ejection fraction (HFrEF), where EF is ≤40% and is primarily due to impaired ventricular contraction, commonly seen in conditions like ischemic heart disease and dilated cardiomyopathy. Heart failure with preserved ejection fraction (HFpEF), with EF ≥50%, is characterized by impaired ventricular relaxation and is often associated with hypertension, obesity, and metabolic syndrome [1,2]. A transitional state between these two categories is heart failure with mildly reduced ejection fraction (HFmrEF), defined by an EF of 41-49% [1,2]. This transitional state offers hope, potentially improving the patient's condition. Thus, even as some patients report improvements due to appropriate management, others experience disease progression. Additionally, heart failure with improved ejection fraction (HFimpEF) refers to patients who previously had HFrEF but have recovered to an EF >40%, typically due to effective treatment.

The AHA and the American College of Cardiology (ACC) also classify HF into four progressive stages [2]. Stage A includes individuals at high risk for HF but without structural heart disease or symptoms, such as those with hypertension or diabetes. Stage B includes patients with structural heart disease but no symptoms of HF, such as those with left ventricular hypertrophy or a history of myocardial infarction. Stage C includes individuals with structural heart disease and past or current HF symptoms requiring medical management. Stage D represents advanced HF, where symptoms persist despite optimal medical therapy, often necessitating specialized interventions like inotropic support, left ventricular assist devices (LVAD), or heart transplantation. This classification helps guide treatment strategies, from lifestyle modifications and risk factor management in early stages to advanced therapies in later stages. Furthermore, the New York Heart Association (NYHA) classified heart failure using a functional classification system that categorizes patients based on their symptoms and physical activity limitations. It consists of four classes: Class I includes patients with no limitation of physical activity and no symptoms with ordinary exertion. Class II describes those with slight limitations, experiencing symptoms like fatigue or dyspnea with ordinary activities but comfortable at rest. Class III involves marked limitation, where even less-than-ordinary activities cause symptoms, though they remain comfortable at rest. Class IV is the most severe, with patients experiencing symptoms even at rest, and any physical activity exacerbates discomfort. This classification is based on the patient's subjective assessment of exertional symptoms, primarily dyspnea, fatigue, and chest discomfort, rather than objective measures like ejection fraction or biomarkers. It is widely used to assess disease progression, guide treatment decisions, and predict prognosis in heart failure management [1,2].

Heart failure affects approximately 6 million American adults, alongside an additional 870,000 persons being diagnosed annually [3]. Presently, heart failure remains the most widespread hospitalization cause in adults aged 65 years and above and has been linked to various physical symptoms that include fatigue, pain, shortness of breath, functional and emotional impairment, fatigue, an increment in caregiver burden, and a reduction in the general quality of life (QoL) [4,5]. Earlier studies have reported higher depression levels in heart failure patients, as well as lower depression treatment levels in such patients [6,7]. It is noteworthy that depression adversely affects HF treatment adherence, as it contributes to poor self-care behaviors and non-compliance to medications, which eventually affect the clinical outcomes [6,7]. Additionally, heart failure has higher mortality rates and a five-year lower survival rate compared to some other morbidities like various types of cancers [8]. For instance, at present, HF mortality rates have surpassed those of prostate and breast cancers [8]. Certain approximations have reported that 50% of heart failure patients are prone to die within five years following diagnosis [9], even as another study has approximated a 36% death rate following a heart failure-associated hospitalization [10]. Still, regardless of the advancements realized in heart failure treatment, it is approximated that 40% of heart failure patients are prone to die within the initial year of their hospitalization [3,11]. The prevalence rate of heart failure has progressively increased over the years and is forecasted to increase by 25% between 2010 and 2030 [9,12].

Normally, heart failure patients characteristically experience devastating emotional and physical symptoms, social role disruptions, and loss of independence, which have an immensely negative impact on the individual’s quality of life [3,13,14]. Moreover, the social determinants of health affect cardiovascular outcomes, particularly in heart failure patients, in several ways. For instance, low socioeconomic status patients always experience fiscal barriers limiting their access to healthcare, delaying diagnosis, and hindering treatment adherence, resulting in worse outcomes for HF patients [3,10-14]. Consequently, inadequate health insurance and geographical disparities have been acknowledged to hinder access to advanced therapies and specialized cardiology interventions, worsening the disease progression [10-14]. Limited education and lower health literacy levels may also lead to mismanagement of symptoms, delays in medical seeking, and increased rates of hospital readmissions. Further, in heart failure patients, environmental factors, including housing instability, food insecurity, and pollution, contribute to increased cardiovascular stress and poor quality of life [10-14]. In advanced heart failure, the physical symptoms, which include shortness of breath, fatigue, progressively worsening dependent edema, and functional decline, have been acknowledged as being immensely distressing to both patients and their caregivers despite remaining largely under-recognized and under-treated [8,11]. As such, heart failure patients and their caregivers are tasked with the challenge of making decisions about end-of-life goals and goals of care, which ultimately will lead to intricate decisions like therapies to improve, sustain, or mitigate worsening, some of which include assistive pumps, life vests, AICDs, cardiac transplantations, other cardiac devices, symptomatic support, and other care plans and decision support [13]. Additionally, the management of heart failure poses an immense fiscal and resource burden on the patients, their families, society, and healthcare systems, with the direct cost of heart failure care forecasted to reach over $77 billion by the year 2030, which represents an increase of 215% concerning the present expenditure [14]. The projected increase in HF care cost is attributable to factors that include rising hospital readmission rates, aging populations, the economic burden associated with the management of chronic diseases, and increased usage of advanced HF therapies [13,14]. In comparison, the United States projected economic burden of diabetes is anticipated to exceed $622 billion by the year 2030, even as Alzheimer’s disease-associated healthcare costs are approximated to surpass $1.1 trillion by 2030 [13-16].

Palliative care (PC) is a specialized medical approach designed to relieve symptoms and enhance the quality of life for patients facing serious, life-threatening illnesses. In the context of cardiovascular disease (CVD), PC plays a crucial role in managing conditions such as heart failure, coronary artery disease, pulmonary arterial hypertension, and valvular heart disease. These conditions not only have a profound impact on a patient’s quality of life but are also often progressive, requiring long-term management and carrying a high risk of mortality. Among these, heart failure stands out due to its unpredictable trajectory, which can range from chronic, manageable stages to advanced, end-stage disease characterized by frequent exacerbations, worsening symptoms, and recurrent hospitalizations. Given this complexity, integrating palliative care into heart failure management is essential, particularly at the end-of-life stage, where symptom burden and psychosocial distress are most pronounced. Palliative care in heart failure goes beyond symptom control; it addresses the holistic needs of patients by providing support for physical, emotional, and psychosocial challenges while also involving shared decision-making to align treatment with the patient’s goals and values. This study aims to explore the intersection of heart failure and palliative care, emphasizing its role in end-of-life practices to improve comfort, dignity, and overall well-being for patients navigating the final stages of the disease [15-17]. 

Moreover, recent studies focusing on palliative care interventions for heart failure patients have associated the palliative care approach with improvements in the patient’s quality of life, significant reductions in the symptom burden, and enhanced caregiver outcomes [18]. Within the HF context, palliative care entails a patient-centered approach that seeks to improve the patient’s quality of life through tackling psychosocial, physical, and spiritual distress. PC is applicable throughout all HF stages and is often integrated with disease-modifying treatments, especially in individuals with higher symptom burdens and intricate care requirements [15-18]. Thus, various clinical guidelines, including those from the AHA, have recommended the earlier integration of PC in advanced HF, even as emergent evidence has supported the benefits of PC during earlier stages of HF [1,2]. Studies have also indicated that PC enhances the quality of life while reducing psychological distress without affecting survival [19,20]. Nevertheless, available evidence for the effectiveness of palliative care originates from oncology, even as the role of palliative care in relation to the management of nonmalignant and chronic conditions, including heart failure, remains underdeveloped [19]. Currently, there are a number of emergent non-oncology palliative care trials in heart failure, including the PAL-HF and ENABLE-HF trials [18,19]. According to Luckett et al., palliative care assumes a number of forms, including primary palliative care and specialty palliative care [20]. Thus, primary palliative care is mainly provided by primary care physicians, nurse practitioners, and cardiologists, while specialty palliative care is provided by multidisciplinary palliative care teams, which include palliative care physicians, social workers, and nurses [2]. Traditionally, palliative care experts work with the primary clinicians of patients to consult and manage the palliative needs of the patients [21]. On the other hand, primary palliative care, including generalist and basic palliative care, implies the concept that every clinician, irrespective of specialization, should have competent basic palliative skills [22]. Such skills include basic management of emotional and physical symptoms, initial discussion of care goals, as well as patient referral to advanced and specialty palliative care for patients in hospice care and at the end-of-life stages. Still, palliative care differs based on aspects of service location. In this systematic review, we evaluate the intersection of heart failure and palliative care as a holistic approach to enhancing the quality of life for heart failure patients.

Regarding the opportunities to integrate palliative domains in heart failure care

Historically, palliative care has often been perceived as a last-resort option, introduced only when curative treatments have failed, creating a false dichotomy between life-prolonging therapies and symptom management. However, modern palliative care frameworks emphasize its integration throughout the disease trajectory, particularly for chronic, progressive illnesses like heart failure. Given the unpredictable course of heart failure, waiting for a defined "trigger" to initiate palliative care can delay essential support that could improve symptom burden, quality of life, and shared decision-making. Instead, a proactive approach incorporating primary palliative care (delivered by cardiologists, heart failure specialists, and primary care providers) and specialty palliative care (for complex cases requiring advanced symptom management and goal setting) ensures comprehensive, patient-centered care. Palliative care should ideally be initiated at diagnosis of advanced heart failure or with early signs of significant symptom burden to ensure timely, patient-centered support, as recommended by ACC, AHA, and HFSA guidelines [1,4,11].

Primary PC interventions in HF deliverable by HF specialists, cardiologists, and primary care physicians include symptom management, advance care planning, provision of spiritual and psychosocial support, and surrogate decision-making, which can be seamlessly integrated into routine cardiology and primary care visits. In contrast, specialty palliative care is particularly valuable in refractory symptoms, conflicting treatment expectations, and complex medical decision-making. For instance, a 65-year-old patient with NYHA Class III HF experiencing refractory symptoms, frequent hospitalizations, elevated levels of anxiety, and family conflicts with regard to advanced treatments should be moved to a specialty PC for comprehensive symptom management and intricate decision-making support. Recognizing that disease severity measures such as ejection fraction may not always align with patient-reported outcomes, the care team's regular assessment of the quality of life, symptom burden, and patient goals is essential. This patient-centered approach ensures that palliative interventions are tailored to individual needs rather than dictated solely by prognosis, ultimately improving overall care and reducing unnecessary hospitalizations. Organizations such as the Center to Advance Palliative Care (CAPC) and the National Hospice and Palliative Care Organization (NHPCO) advocate for early integration of palliative care in heart failure management, reinforcing that it should complement, rather than replace, disease-modifying therapies (CAPC, NHPCO) [23-25].

Additionally, palliative care (PC) is a recommended standard of care for all heart failure patients, with substantial evidence supporting its role in improving patient outcomes, reducing symptom burden, and enhancing shared decision-making [26]. Studies from AHA, ACC, and the Heart Failure Society of America (HFSA) emphasize that early integration of palliative care in heart failure management significantly improves the quality of life, reduces hospitalizations, and ensures that care aligns with patient values and goals. Furthermore, PC helps address healthcare disparities, particularly among marginalized populations who may face barriers to optimal heart failure management [26].

Despite its benefits, widespread implementation of palliative care remains challenging due to the limited availability of specialized palliative care clinicians [1,21,22,26,27]. Given this shortage, the ACC and AHA recommend a tiered approach, where primary palliative care, including symptom management and goal-directed discussions, is provided by cardiologists, heart failure specialists, and primary care physicians, while specialty palliative care is reserved for complex cases requiring advanced symptom control, psychosocial support, and end-of-life care planning. Current research continues to explore the most effective strategies for integrating palliative care into heart failure treatment, particularly in managing heart failure with reduced ejection fraction (HFrEF), where symptom burden is high, and disease progression can be unpredictable. Integrating palliative care into cardiology requires a multidisciplinary approach involving physicians, nurses, pharmacists, social workers, and chaplains to ensure comprehensive, patient-centered care [23]. This model facilitates the management of distressing symptoms such as dyspnea, fatigue, depression, and anxiety while fostering ongoing discussions to align treatment with the patient’s evolving goals and preferences. The multidisciplinary team, comprising nurses, pharmacists, physicians, chaplains, and social workers, is tasked with the crucial role of optimizing symptom management, offering spiritual and psychosocial support, and tackling social determinants of health to ascertain comprehensive PC in HF. Further, it is worth noting that, currently, several barriers have been acknowledged to contribute to the observed disparities in palliative care and include socioeconomic limitations, ethnic and racial disparities, inadequate provider training, geographic inequities, family and patient misconceptions regarding the equation of PC to end-of-life care, and reimbursement challenges [26,27]. Some of the notable strategies for overcoming such barriers to PC integration include the expansion of training for cardiologists, the use of telemedicine for underserved populations, the development of community-based programs, and the increment of public awareness on the benefits of PC.

This systematic review evaluates how integrating palliative care into heart failure management enhances patient quality of life by addressing symptom control, improving care satisfaction, and ensuring informed decision-making throughout the disease trajectory. A holistic approach that incorporates symptom management, emotional and spiritual support, and early advance care planning is critical in optimizing care, particularly for those with advanced heart failure. Through a review of existing literature, this study seeks to contribute to ongoing efforts in optimizing end-of-life care delivery, clarifying best practices for integrating palliative care into heart failure management, and reinforcing its role in improving patient and family well-being.

## Review

Materials and methods

An extensive literature search was conducted using online databases, including Embase, PubMed, SCOPUS, Web of Science, and Google Scholar. The selected references included health assessments, epidemiological studies with anonymized data, published reviews, and multi-center studies. Further, the identification and removal of duplicate references were done through a combination of manual cross-verification and EndNote reference management software. Thus, for references with similar populations and study periods, in cases where two or more publications reported on datasets that overlapped, a cautious review of their methodologies, data completeness, sample sizes, and relevance to the study objectives was conducted. In instances where overlapping was identified, the reference that offered more comprehensive and pertinent data, including detailed outcome reporting and bigger sample sizes, as well as higher methodological quality, was retained. This was mainly performed to minimize the duplication of data and avert over-representation of similar populations. 

Literature search strategy

The search strategy for relevant studies involved thoroughly exploring several medical databases, including Embase, PubMed, SCOPUS, Web of Science, and Google Scholar. To find relevant references, the approach combined various Medical Subject Headings (MeSH) keywords such as “advance care planning,” “quality of life,” “symptom burden,” “heart failure,” and “palliative care,” utilizing different Boolean operators, namely "AND," "OR," and “NOT." This comprehensive search yielded a total of 951 references.

Additionally, the search strategy encompassed two phases and used the preferred reporting items for systematic reviews and meta-analyses (PRISMA) guidelines for the selection and inclusion of articles. The initial phase involved the performance of an independent screening of the title and abstract of every retrieved article by two researchers. In case of inadequate data in the study's abstract to inform its retention or exclusion decision, the exclusion decision was only arrived at following full-text screening. Nonetheless, articles with insufficient abstract data but with pertinent titles to the systematic review were included in the second phase that entailed full-text screening. The second phase involved the full-text screening of the retained articles for the study inclusion and exclusion criteria. All potential disputes were resolved through a third researcher tasked with making a final decision on whether a disputed article should be included or excluded, and the decision was mainly arrived at via consensus and consultations.

Study inclusion and exclusion criteria

For this review, the criteria for inclusion encompassed crossover design studies, randomized controlled trials (RCTs), systematic reviews, and meta-analyses, as well as prospective cohort studies that met the following specifications: studies focusing on heart failure treatment and palliative care, published in English and conducted between the years 2000 and 2025. Conversely, the criteria for exclusion comprised sponsored clinical trials, opinion pieces, editorials, narrative reviews, dissertations, studies initially published in languages other than English, and studies published in non-peer-reviewed journals. Furthermore, the extraction of essential data from the included studies was executed as follows: (a) general study attributes, including the names of the authors, the year of the study, publication, and the sampling methods utilized; (b) attributes of the study population, such as ethnicity and race, sample size, nationality, age, and gender of study participants, as well as follow-up measures; (c) the type and duration of the intervention; and (d) the principal findings of the study.

Results

**Figure 1:**
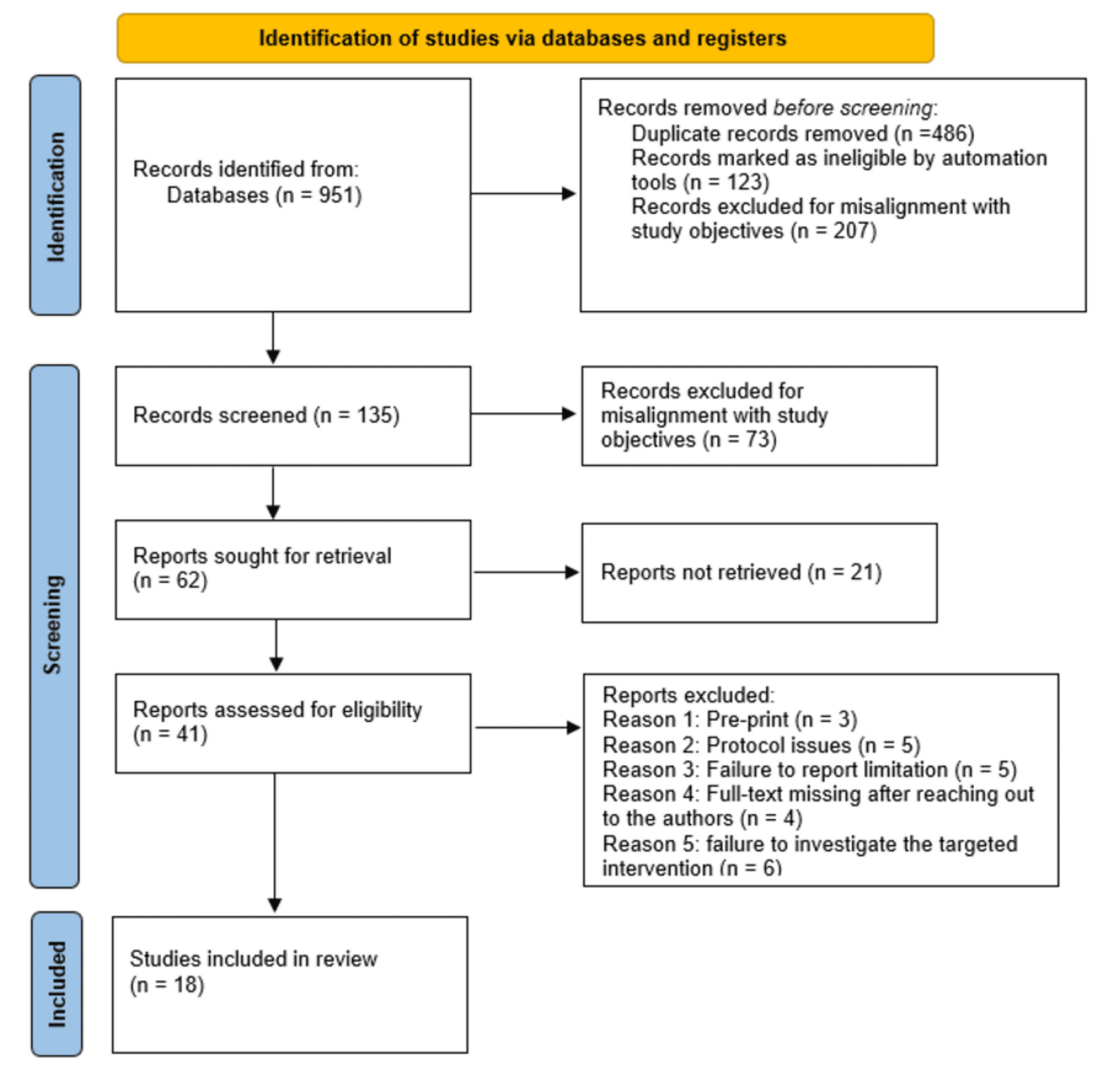
PRISMA flow diagram indicating the study selection process for this systematic review

The selection process for studies in this systematic review followed the preferred reporting items for systematic reviews and meta-analyses (PRISMA) guidelines. Consequently, 951 references were retrieved following a comprehensive literature search across various online databases. The initial screening of these references resulted in identifying and removing 486 duplicates, while an additional 123 references were deemed ineligible through automated processes. Furthermore, an additional 207 references were excluded for various reasons, including alignment discrepancies with the objectives of this systematic review. As a result, 135 studies fulfilled the inclusion criteria established and were subject to further scrutiny, culminating in the exclusion of 73 studies. The remaining 62 studies were pursued for retrieval, of which 21 were determined to be irretrievable. Thus, 41 studies underwent additional evaluations for eligibility, resulting in the exclusion of 23 studies post full-text screening for reasons that included protocol issues (five studies), availability of preprints (three studies), missing full text despite communication with the authors (four studies), failure to report limitations (five articles), and inadequate investigation of the targeted intervention (six studies). Ultimately, 18 studies satisfied the inclusion criteria, qualifying for incorporation into this systematic review. These studies were then assessed and discussed in conjunction with the findings of other studies to substantiate the study's conclusions [17-53]. The PRISMA flow diagram in Figure 1 illustrates the study selection process employed in this systematic review.

The studies selected and included in this systematic review have been summarized in Table 1. 

**Table 1:** Summary of the included studies, study designs, and their findings

Author name/Citation	Study design	Sample size	Findings
Morrison RS, Dietrich J, Ladwig S, et al. [17]	Retrospective cohort study	1717	The study disclosed that multidisciplinary palliative care consultation teams led to significant reductions in hospital costs for Medicaid beneficiaries in addition to improving the patients' quality of life, and this was attributed to increased hospice referrals.
Wong CY, Chaudhry SI, Desai MM, Krumholz HM [28]	Observational study	1395	Significant percentages of heart failure patients tend to experience several comorbidities, and functional limitations, in addition to being prescribed several medications, aspects that contribute to the complex nature of heart failure treatment and management. This highlights the significance of an inclusive approach to heart failure care, which addresses complex challenges experienced by the patients.
Liu Y, Tao L, Liu M, et al. [29]	Randomized controlled trial (RCT)	103	The study found that palliative care resulted in significant improvements in the physical and mental status of the patients, as well as the general quality of life, particularly in patients with chronic heart failure.
Çamcı G, Oğuz S [30]	Randomized controlled trial (RCT)	84	The study has disclosed that patients with severe heart failure receiving palliative care experienced enhanced symptom management during the initial month following hospitalization and reported significantly lower rates of re-hospitalization at 1, 3, and 6 months.
Balata M, Radbruch L, Hesse M, et al. [31]	Multicenter, parallel, two-arm, open-label randomized controlled trial (RCT)	843	The study has disclosed that timely integration of palliative care in heart failure management results in significant improvement in the patients' quality of life and symptom burden in comparison to standard cardiac care.
McKelvie RS, Moe GW, Cheung A, et al. [33]	Clinical practice guideline	Not applicable	The study emphasized the significance of acknowledging and managing sleep apnea in patients with chronic heart failure, tackled the different challenges brought about by renal dysfunction, discussed the function of mechanical circulatory support for advanced heart failure instances, and emphasized the importance of integrating palliative care into heart failure care to enhance patient outcomes.
Temel JS, Greer JA, Muzikansky A, et al. [34]	Randomized controlled trial (RCT)	151	The study disclosed that integration of early palliative care with oncologic treatment resulted in significant improvements in the patient’s quality of life and mood, considerably reduced belligerent end-of-life care, in addition to bringing about prolonged median survival in comparison to standard care only.
Mirshahi A, Bakitas B, Khoshavi M, et al. [35]	Randomized Controlled Feasibility Study (RCT)	50	The study found that the integration of early palliative care telehealth interventions resulted in improvements in the quality of life of heart failure patients, indicating the feasibility and possible advantages for the management of symptoms and well-being.
Smith TJ, Coyne P, Cassel B, et al. [36]	Retrospective cohort study	237	The study disclosed that the implementation of high-volume specialist palliative care units with a devoted interdisciplinary team alongside standardized care protocols resulted in significant reductions in everyday hospital fees and overall costs for severely ill patients, indicating that such units are capable of lowering the expenses associated with in-hospital end-of-life care.
Norton SA, Hogan LA, Holloway RG, et al. [37]	Retrospective cohort study	191	For the high-risk patients in intensive care units, proactive palliative care consultations resulted in significant reductions in the length of stay from 16.28 days to 8.96 days, devoid of impacting discharge dispositions and mortality rates.
Sidebottom AC, Jorgenson A, Richards H, Kirven J, Sillah A. [40]	Randomized controlled trial (RCT)	232	For inpatient acute heart failure patients, inpatient palliative care services resulted in significant temporary improvements in the quality of life, symptom burden, and depressive symptoms.
Hopp FP, Zalenski RJ, Waselewsky D, et al. [41]	Quasi-experimental study	85	The study found that 9.3% of individuals receiving palliative care consultations chose comfort-oriented care, in comparison to none within the normal care group; nonetheless, the observed differences were not statistically significant.
Schellinger S, Sidebottom A, Briggs L [42]	Implementation study/Quality improvement study	1894	The study has disclosed that participants are more liable to have completed different health directives and used palliative care services than non-participants.
Ng AY, Wong FK [44]	Randomized controlled trial (RCT)	84	The study disclosed that a 12-week home-based palliative care for heart failure patients significantly improved quality of life, in addition to improving patient satisfaction levels even as it reduced the caregiver burden in end-stage heart failure patients.
Khajehpoor MH, Shahrbabaki PM, Nouhi E [45]	Randomized controlled trial (RCT)	100	The study disclosed that home-based palliative care programs resulted in significant improvements in elderly heart failure patients’ quality of life.
Brännström M, Boman K [46]	Randomized controlled trial (RCT)	36	The study has disclosed that the integration of patient-centered palliative care with standard heart failure treatment and management resulted in significant improvements in health-related quality of life, as well as reductions in hospitalizations for severe chronic heart failure patients.
Santos KA, Trotte LMC, Telles A, et al. [51]	Qualitative descriptive study	10	The study disclosed that the integration of palliative care into heart failure management should be facilitated by specialized multidisciplinary teams based on institutional protocols. Though the use of palliative care in heart failure management leads to improvement in patient’s quality of life, the integration of palliative care into heart failure management is mainly hindered by challenges that include patient profiles, systemic organizational issues, and emotional burdens on providers.
Bekelman DB, Plomondon ME, Carey EP, et al. [53]	Randomized controlled trial (RCT)	392	The study disclosed that the patient-centered disease management (PCDN) approach for heart failure care did not have significant improvement in patient health status in comparison to normal care, despite its association with reduction in mortality and improvement of depression outcomes.

Risk of bias (RoB) assessment

A risk of bias (RoB) assessment was conducted to evaluate the internal validity of the studies included in this analysis. The primary objective of performing a RoB assessment is to ascertain whether the findings of these studies were subject to various biases that could potentially lead to an over- or under-estimation of the effects of the intervention. Consequently, this study utilized the Assessment Tool for Systematic Reviews (AMSTAR) 2. The domains employed to assess the quality of the included studies encompass reporting bias, detection bias, confounding bias, and selection bias. Thus, the reporting bias was evaluated through the determination of whether the results were selectively reported and whether there were protocols for comparison. Consequently, detection bias was assessed based on the binding procedures and outcome measures objectivity. Still, the confounding bias was assessed through the evaluation of how the studies accounted for major covariates and if sufficient adjustments were implemented in statistical analyses. Also, the selection bias was evaluated through the analysis of the study inclusion criteria, randomization methods (whenever applicable), as well as the potential baseline differences between different groups. The results from the RoB assessment are documented in Table 2.

**Table 2:** Risk of bias assessment for the included studies

Author name/Citation	Study design	The overall risk of bias
Morrison RS, Dietrich J, Ladwig S, et al. [17]	Retrospective cohort study	Moderate
Wong CY, Chaudhry SI, Desai MM, Krumholz HM [28]	Observational study	Moderate
Liu Y, Tao L, Liu M, et al.	Randomized controlled trial (RCT)	Low
Çamcı G, Oğuz S [30]	Randomized controlled trial (RCT)	Low
Balata M, Radbruch L, Hesse M, et al. [31]	Multicenter, parallel, two-arm, open-label randomized controlled trial (RCT)	Low
McKelvie RS, Moe GW, Cheung A, et al. [33]	Clinical practice guideline	Low
Temel JS, Greer JA, Muzikansky A, et al. [34]	Randomized controlled trial (RCT)	Low
Mirshahi A, Bakitas B, Khoshavi M, et al. [35]	Randomized Controlled Feasibility Study (RCT)	Moderate
Smith TJ, Coyne P, Cassel B, et al. [36]	Retrospective cohort study	Moderate
Norton SA, Hogan LA, Holloway RG, et al. [37]	Retrospective cohort study	Moderate
Sidebottom AC, Jorgenson A, Richards H, Kirven J, Sillah A. [40]	Randomized controlled trial (RCT)	Low
Hopp FP, Zalenski RJ, Waselewsky D, et al. [41]	Quasi-experimental study	Low
Schellinger S, Sidebottom A, Briggs L [42]	Implementation study/Quality improvement study	Low
Ng AY, Wong FK [44]	Randomized controlled trial (RCT)	Low
Khajehpoor MH, Shahrbabaki PM, Nouhi E [45]	Randomized controlled trial (RCT)	Low
Brännström M, Boman K [46]	Randomized controlled trial (RCT)	Low
Santos KA, Trotte LMC, Telles A, et al. [51]	A qualitative descriptive study.	Low
Bekelman DB, Plomondon ME, Carey EP, et al. [53]	Randomized controlled trial (RCT)	Low

A measurement tool to assess systematic reviews (AMSTAR 2) assesses studies based on 16 questions. These questions have been listed as follows.

1. Did the research questions and inclusion criteria for the review include the components of PICO?

2. Did the report of the review contain an explicit statement that the review methods were established before the conduct of the review, and did the report justify any significant deviations from the protocol?

3. Did the review authors explain their selection of the study designs for inclusion in the review?

4. Did the review authors use a comprehensive literature search strategy?

5. Did the review authors perform study selection in duplicate?

6. Did the review authors perform data extraction in duplicate?

7. Did the review authors provide a list of excluded studies and justify the exclusions?

8. Did the review authors describe the included studies in adequate detail?

9. Did the review authors use a satisfactory technique for assessing the risk of bias in individual studies that were included in the review?

10. Did the review authors report on the sources of funding for the studies included in the review?

11. If meta-analysis was performed, did the review authors use appropriate methods for statistical combination of results?

12. If meta-analysis was performed, did the review authors assess the potential impact of risk of bias in individual studies on the results of the meta-analysis or other evidence synthesis?

13. Did the review authors account for the risk of bias in primary studies when interpreting/discussing the results of the review?

14. Did the review authors provide a satisfactory explanation for, and discussion of, any heterogeneity observed in the results of the review?

15. If they performed quantitative synthesis, did the review authors carry out an adequate investigation of publication bias (small study bias) and discuss its likely impact on the results of the review?

16. Did the review authors report any potential sources of conflict of interest, including any funding they received for conducting the review?

Additionally, the ratings are further defined as follows: High: No or a single non-critical weakness. Moderate: Over one non-critical weakness without any critical weakness. Low: One or several critical weaknesses. The findings of the AMSTAR 2 assessment have been presented in Table 3.

**Table 3:** AMSTAR 2 assessment of the included studies Y: Yes. N: No. AMSTAR: A measurement tool to assess systematic reviews

Author name/Citations	Q1	Q2	Q3	Q4	Q5	Q6	Q7	Q8	Q9	Q10	Q11	Q12	Q13	Q14	Q15	Q16	Overall rating
Morrison RS, Dietrich J, Ladwig S, et al. [17]	Y	Y	Y	Y	N	Y	Y	Y	Y	N	Y	Y	Y	Y	N	Y	Moderate
Wong CY, Chaudhry SI, Desai MM, Krumholz HM [28]	Y	N	Y	Y	N	N	Y	Y	Y	N	Y	N	Y	Y	N	Y	Low
Liu Y, Tao L, Liu M, et al. [29]	Y	Y	Y	Y	Y	Y	Y	Y	Y	Y	Y	Y	Y	Y	Y	Y	High
Çamcı G, Oğuz S [30]	Y	Y	Y	Y	Y	Y	Y	Y	Y	Y	Y	Y	Y	Y	Y	Y	High
Balata M, Radbruch L, Hesse M, et al. [31]	Y	Y	Y	Y	Y	Y	Y	Y	Y	Y	Y	Y	Y	Y	Y	Y	High
McKelvie RS, Moe GW, Cheung A, et al. [33]	Y	N	Y	Y	N	N	Y	Y	Y	N	Y	N	Y	Y	N	Y	Low
Temel JS, Greer JA, Muzikansky A, et al. [34]	Y	Y	Y	Y	Y	Y	Y	Y	Y	Y	Y	Y	Y	Y	Y	Y	High
Mirshahi A, Bakitas B, Khoshavi M, et al. [35]	Y	Y	Y	Y	Y	Y	Y	Y	Y	Y	Y	Y	Y	Y	Y	Y	High
Smith TJ, Coyne P, Cassel B, et al. [36]	Y	N	Y	Y	N	N	Y	Y	Y	N	Y	N	Y	Y	N	Y	Low
Norton SA, Hogan LA, Holloway RG, et al. [37]	Y	N	Y	Y	N	N	Y	Y	Y	N	Y	N	Y	Y	N	Y	Low
Sidebottom AC, Jorgenson A, Richards H, Kirven J, Sillah A. [40]	Y	Y	Y	Y	Y	Y	Y	Y	Y	Y	Y	Y	Y	Y	Y	Y	High
Hopp FP, Zalenski RJ, Waselewsky D, et al. [41]	Y	Y	Y	Y	Y	Y	Y	Y	Y	Y	Y	Y	Y	Y	Y	Y	High
Schellinger S, Sidebottom A, Briggs L [42]	Y	N	Y	Y	N	N	Y	Y	Y	N	Y	N	Y	Y	N	Y	Low
Ng AY, Wong FK [44]	Y	Y	Y	Y	Y	Y	Y	Y	Y	Y	Y	Y	Y	Y	Y	Y	High
Khajehpoor MH, Shahrbabaki PM, Nouhi E [45]	Y	Y	Y	Y	Y	Y	Y	Y	Y	Y	Y	Y	Y	Y	Y	Y	High
Brännström M, Boman K [46]	Y	Y	Y	Y	Y	Y	Y	Y	Y	Y	Y	Y	Y	Y	Y	Y	High
Santos KA, Trotte LMC, Telles A, et al. [51]	Y	N			N	N				N		N			N	Y	Low
Bekelman DB, Plomondon ME, Carey EP, et al. [53]	Y	Y	Y	Y	Y	Y	Y	Y	Y	Y	Y	Y	Y	Y	Y	Y	High

Quality assessment

The appraisal tool for cross-sectional studies (AXIS), a critical assessment tool consisting of 20 items, was utilized to evaluate the quality of the included studies. To accomplish this, three independent reviewers were assigned to assess the quality of each study, resolving any disagreements through discussions and consensus. Furthermore, each study was scored as 1 (yes) or 0 (no), with “don’t know” for inapplicable items. The ‘don’t know’ response in the AXIS tool was treated as equal to a "no" response and was scored 0 to ensure the maintenance of an increasingly conservative approach in the quality assessment. The calculation of each study's overall quality was performed through the addition of the overall amount of "yes" responses (with a score of 1) and subsequently divided by the overall amount of applicable items. Further, the studies were categorized as high quality if they attained a score of ≥75% and moderate quality if they attained a score of between 50% and 74%. In this systematic review, no study was categorized as low quality (score <50%). Overall, the quality of the included studies ranged from moderate to high, with six studies classified as moderate quality while the remainder were of high quality.

Data extraction

The authors employed a data extraction form to obtain pertinent data from the diverse studies included in the analysis. Furthermore, information regarding various study attributes, such as author names, publication year, study design, sample sizes, and findings, was systematically extracted from each study. Consequently, three independent reviewers were responsible for extracting the data, and any discrepancies were addressed through discussion and consensus. Thus, to ascertain reliability and consistency in data extraction, an assessment of the inter-rater agreement was conducted using Cohen’s kappa statistic (κ = 0.82), and potential disagreements were mainly resolved through discussion and adjudication by the third reviewer. The included study attributes were primarily based on their pertinence to the objectives of the study and prior systematic reviews, ascertaining comparability, and methodological rigor across the included studies.

Discussion

Providing care for heart failure patients is intricate and challenging, as the care is affected by several cognitive, physical, environmental, and social factors [28]. The various disease stages also influence the direction of care. For instance, the severity of heart failure, as classified by the NYHA functional system, determines the appropriateness of guideline-directed medical therapy (GDMT), device-based therapies, and palliative care integration. In early-stage heart failure (NYHA Class I), GDMT is the foundation, including renin-angiotensin system inhibitors (ACE inhibitors, ARBs, or ARNIs), beta-blockers, SGLT2 inhibitors, and magnetic resonance angiographies (MRAs) for HFrEF. ICDs or CRTs are not typically indicated unless there is a specific arrhythmic or conduction abnormality. At this stage, PC focuses on education, advanced care planning, and symptom management for high-risk patients. As symptoms progress (NYHA Class II-III), guideline-directed medical therapy (GDMT) remains essential, with ICDs recommended for sudden cardiac death prevention in symptomatic HFrEF (LVEF ≤35%) and CRT for patients with QRS ≥150 ms and left bundle branch block (LBBB) to improve symptoms and survival. Palliative care plays a supportive role by addressing symptom burden and psychosocial needs and aligning treatment goals with patient preferences, particularly in those with worsening quality of life despite optimized GDMT. In advanced heart failure (NYHA Class IV), GDMT may be limited by hypotension, worsening renal function, or frailty, requiring careful medication adjustments. Device therapy (ICDs/CRT) is used selectively, with emphasis on LVADs, heart transplantation, or shifting to palliative care and hospice for end-stage patients. In this regard, PC plays an increasingly proactive role in HF management by guiding intricate decision-making, alleviating the disease symptoms, as well as ascertaining that the treatment is aligned with the goals of the patient during the course of the disease [4,29-32].

As the disease progresses to the end-of-life phase, managing emotional, spiritual, and physical symptoms becomes crucial alongside advance care planning. Patients with heart failure at this stage report a higher burden of symptoms, including fatigue, dyspnea, depression, edema, anxiety, confusion, anorexia, insomnia, and poor quality of life [29,30]. Despite this, the prognosis for heart failure patients remains unpredictable, with their care preferences varying in quality and quantity [4]. This highlights the importance of a patient-centered care approach in advance care planning. However, recent studies indicate that there is an insufficient level of individualized education and effective communication regarding heart failure care [4]. Given the complex nature of heart failure management, the inadequacy of effective communication about treatment options, and the importance of patient-centered planning, it is evident that integrating palliative care into heart failure management is appropriate [4,30,31]. However, dissimilar to cancer, HF is marked by unpredictable courses with periodic exacerbations, prognostication challenges, and sudden hospitalizations, which makes the provision of end-of-life care planning increasingly intricate. Various studies have revealed that the symptom burden associated with heart failure is comparable to, and in some cases may even exceed, that experienced by cancer patients [32,33]. Similar to cancer, the significant symptom burden of heart failure encompasses spiritual, caregiver, and emotional stress, along with an uncertain prognosis. Surveys conducted with palliative care (PC) teams, particularly within cancer populations, have revealed numerous benefits, including enhancements in patients’ quality of life, survival rates, anxiety levels, pain management, and moods [33-35]. Furthermore, PC has been associated with reduced costs, lower hospitalization rates, and increased patient satisfaction across various populations [34,36-38]. The relevance of PC, along with its potential benefits for patients with advanced heart failure, has been recognized by the Heart Failure Society of America (HFSA) and the American Heart Association (AHA). Thus, the benefits of PC in the reduction of costs, hospitalizations, and improvement of satisfaction have been shown in both HF and cancer populations [23].

Nevertheless, despite the guidelines and consensus panels advocating for the simultaneous delivery of PC alongside heart failure life-prolonging treatments for patients and the AHA’s recent recommendations to refer advanced heart failure patients to PC [13], a limited consensus has been reached on specific practices. Therefore, conducting a systematic review of the impact of PC interventions on heart failure care outcomes, particularly regarding patients’ quality of life, will help shape effective practice recommendations and policy frameworks. Moreover, due to the global burden of heart failure, it is urgent to understand the best methods for integrating palliative care to alleviate the strain on health and human resources [39]. A limited number of studies have established major interventional approaches for incorporating PC into heart failure treatment services.

A study by Sidebottom et al. effectively compared the effects of inpatient consultations from the palliative care (PC) team for heart failure patients to standard care for hospitalized acute heart failure patients [40]. The findings revealed statistically significant improvements in quality of life, mood, and symptom burden, despite no significant effect on patient survival. However, despite the correlation between the PC integration intervention and increased advance care planning, the study found no impact on 30-day readmission or hospice referrals [40]. Factors that include late PC initiation, patient population heterogeneity, and the probability of PC interventions often tend to improve the quality of life as opposed to the survival outcomes. Similar results were noted in a study by Hopp et al., which evaluated the effect of integrating inpatient PC consultations into heart failure care in US hospitals, focusing on areas such as symptom assessment and management, care goals elicitation, discharge planning, and advance care planning [41].

Aside from playing a crucial role in addressing specific symptom burdens, PC has also been recognized for its ability to inspire various care goals and assist patients and caregivers in complex decision-making situations [17]. In this context, Schellinger et al. argue that advance care planning (ACP) is an effective tool for heart failure patients, aiding them in these processes [42]. Moreover, referral to ACP constitutes one of the standard discharge procedures for heart failure patients, although it may not be sufficient to encourage participation without discussing the process. Consequently, studies have shown that heart failure patients were increasingly likely to complete ACP processes after discharge, primarily due to conversations about the ACP process and recommendations from the PC provider [17,37,42,43]. Completing the ACP process primarily focuses on enabling proxies to make decisions per the patient's preferences and subsequently documenting these preferences in medical records, often achieved through a standard advance directive. Completing ACP processes has also been associated with a greater use of hospice services during end-of-life stages [42].

In their study, Ng and Wong revealed that home-based palliative care programs, which comprised multidisciplinary teams of nurses, physicians, spiritual care providers, and social workers, were effective, as they significantly improved the quality of life for heart failure patients by offering effective symptom management, advance care planning, psychosocial support, and caregiver support [44]. This enhancement was evident in all components of quality of life for patients in the intervention group immediately following the intervention, compared to the pre-intervention stage [44]. Additionally, a significant difference was observed in heart failure patients' quality of life scores in the control group. However, the changes were less pronounced than those in the intervention group [45]. These findings support the results of studies conducted by Brännström et al. and Hosseini et al., which indicated that heart failure patients receiving home-based palliative care experienced an immediate improvement in their quality of life [46,47].

Additionally, the study conducted by Greener et al. revealed that palliative care (PC) could enhance the quality of life for heart failure patients by alleviating various symptoms of the condition, including fatigue, dyspnea, depression, edema, anxiety, confusion, anorexia, insomnia, and poor quality of life [48]. Likewise, the research by Isenberg et al. indicated that, in most cases, even minimal use of PCs led to lower hospital mortality rates and improved experiences for heart failure patients during end-of-life stages [49]. It is noteworthy that, within their study context, Isenberg et al. utilized the phrase "minimal use" to imply patients who reported having had at minimum one PC consultation devoid of prolonged and continuous follow-ups, contrary to those who had sustained and integrated PC services. Such limited engagements have been linked to improvements in hospital outcomes and patient experience during the provision of end-of-life care [49]. Furthermore, the study compared individuals who received home-based PC services with those who did not in the final three months, revealing that for patients with advanced heart failure, home-based PC increased the likelihood of dying in preferred locations while reducing hospital admissions [50].

Regarding outpatient specialty PC, the growing evidence of the benefits of integrating palliative care into heart failure treatment can be drawn from the recent PAL-HF (palliative care in heart failure) study [33]. In the study, the "usual care" group was offered standard cardiology care based on clinical guidelines and symptom management by the cardiologist, even though the care did not include the various structured PC components like interdisciplinary support, spiritual and emotional assessments, and advance care planning [33]. Further, the study randomized 150 heart failure patients at a higher risk of re-hospitalization and six-month mortality to either six months of interdisciplinary palliative care provided by a palliative care-specialized nurse or usual care. The goal of the structured intervention was to enhance the patient's quality of life by addressing all emotional and physical symptoms, advance care planning, and spiritual concerns. Compared to usual care interventions, it was observed that palliative care was associated with clinically significant improvements in heart failure-specific and disease-generic quality of life at six-month follow-ups [33]. The study also revealed statistically significant improvements in various secondary outcomes, including spiritual well-being and mood, although no association was found between palliative care and re-hospitalization or mortality [33]. The Functional Assessment of Chronic Illness Therapy-Spiritual Well-Being Scale (FACIT-Sp) was employed by the study in the assessment of spiritual well-being and the Hospital Anxiety and Depression Scale (HADS) for the evaluation of mood outcomes [33].

Consequently, in the context of home-based specialty palliative care, two distinct studies focused on home-based palliative care interventions that recruited heart failure patients with advanced disease (New York Heart Association Class III-IV) and subsequently provided them palliative care integrated within the broader disease management framework, which included a multidisciplinary team approach and care coordination [40, 51]. The study conducted by Brännström et al., where palliative care was incorporated into home-based heart failure management delivered by a multidisciplinary team, indicated that, compared to usual care interventions, heart failure patients who received palliative care reported clinically significant improvements in their quality of life at six weeks, despite palliative care not having any effect on the burden of their symptoms [46]. Thus, even as the symptom burden remained unchanged, patients reported significant improvements in coping mechanisms, enhanced emotional well-being, and improvement in communication with healthcare providers that collectively contributed to the perceived improvement in the quality of life [46]. Additionally, the study revealed that integrating palliative care into heart failure management led to fewer re-hospitalizations during the six-month study period, even though no significant correlations were found regarding overall care costs [46]. The fewer re-hospitalizations can be attributed to various factors, including the comparatively shorter follow-up duration (six months), the patients' advanced disease stages alongside higher-burden symptoms, as well as the probability of the PC interventions mainly targeting patient-reported outcomes as opposed to the clinical endpoints that include mortality and hospitalization.

In concordance with the findings mentioned above, the research by Sidebottom et al. asserted that a greater proportion (39%) of heart failure patients receiving palliative care experienced substantial enhancements in the New York Heart Association functional classification after six months [40]. Furthermore, the study revealed statistically significant advancements in quality of life within the intervention groups at one- and three-month intervals. Consequently, within the intervention group, quality of life scores improved by approximately 12.92 points compared to the 8 points recorded for the control group [40]. Additionally, Sidebottom et al. reported notable improvements in symptom burden, with a score of 8.39 for the study intervention group compared to 4.7 points documented for the control group [40]. Thus, in the study conducted by Sidebottom et al., the control group indicated a 20% improvement rate in comparison to 39% realized in the intervention group [40]. Additionally, the study evaluated the quality of life (QoL) scores using the Kansas City Cardiomyopathy Questionnaire (KCCQ) [40].

Moreover, the study by Wong et al. focused on transitional palliative care, randomizing 84 heart failure patients discharged from various hospitals. Patients received either palliative care with telephone check-ins and home visits or attention controls involving social phone calls on unrelated topics to heart failure care [52]. The study revealed that at a three-month follow-up, heart failure patients in the intervention groups (who received palliative care) reported significantly fewer re-hospitalizations than those in the control groups and expressed higher satisfaction levels with their care [52]. After three months, the palliative care intervention was associated with clinically significant reductions in symptom burden and improved quality of life [52]. Nonetheless, concerning primary palliative care, the investigation conducted by Bekelman et al. assessed the efficacy of palliative care that included telemonitoring interventions and collaborative care management as opposed to conventional heart failure care [53]. This multisite study, encompassing 392 heart failure patients with diminished quality of life, pronounced symptoms, and restricted functional capacities, revealed that the implementation of a multifaceted palliative care intervention did not yield a statistically significant improvement concerning the primary outcomes (patient health status) in heart failure patients when compared to the outcomes associated with standard heart failure care [53]. The lack of statistically significant primary outcome improvements is attributable to difficulties with regard to patient engagement, intervention delivery variability, advanced HF complexities, and various unmeasured confounders, including caregiver support and socioeconomic aspects. However, notable enhancements in the patients' health status were observed over time within both the intervention and normal care groups; additionally, within the secondary outcomes of the study, there was a reported decrease in mortality rates within the intervention group, along with considerable reductions in depressive symptoms among heart failure patients who exhibited signs of depression, indicating potential survival benefits of PC [53]. Among the patients who were screened and found positive for depression at the baseline, the study reported statistically significant improvements with regard to depressive symptom scores within the intervention group in comparison to the usual care group [53]. Such secondary findings corroborate those of earlier studies that have reported positive impacts of PC and multidisciplinary management on HF patients' psychological well-being and survival.

Study limitation

This study is a humanized literature review, with a key limitation of potential for selection bias and other forms of publication biases arising from the set inclusion criteria. Particularly, the researchers only considered peer-reviewed studies published in English, which might have resulted in the exclusion of pertinent studies published in other notable languages. Moreover, some of the included studies are retrospective cohort studies and qualitative studies in the study limitations section, which have a higher risk of bias. Regardless of such limitations, the bias has been mitigated through the performance of a comprehensive and in-depth search across different databases and complying with the predefined inclusion and exclusion criteria. Additionally, variations in study designs, sample sizes, and methodologies across the included studies limit the generalizability of findings. Relating secondary data from diverse sources may also introduce heterogeneity in outcome measures, making direct comparisons challenging. Furthermore, while our systematic approach adhered to PRISMA guidelines, excluding certain study types, such as qualitative research and case reports, may have restricted a more nuanced understanding of patient experiences with palliative care in heart failure.

## Conclusions

In addition to the evolving evidence supporting PC integration in HF management, these recent studies underscore its growing significance in improving patient outcomes, emphasizing the urgent need for PC integration to alleviate symptom burden, enhance quality of life, and optimize care delivery. Our review supported the growing body of evidence that PC interventions significantly reduce hospitalizations, improve symptom control, and enhance patient and caregiver satisfaction, mainly when introduced early. However, challenges such as limited specialist availability, inconsistent referral patterns, and lack of standardized implementation persist. Evidence suggests that multidisciplinary PC models, including home-based and telehealth interventions, effectively alleviate symptom distress and facilitate advance care planning, aligning treatment with patient goals. Targeted training interventions for the cardiology and generalist teams will equip them with basic PC skills, thereby mitigating the limitations resulting from shortages in specialists. Moreover, standardized referral criteria, integration of telehealth-based PC models, and screening tools for timely identification of PC requirements are essential to improving access to PC and reducing care delivery disparities. While PC does not universally impact survival, its role in reducing healthcare utilization, addressing psychosocial distress, and ensuring goal-concordant care is well established. Given the growing body of research advocating for PC in HF, structured guidelines, including referral criteria, integration into HF guidelines, and the timing of introduction, as well as broader clinical adoption, are necessary to maximize its benefits in this high-burden population.
